# Association Between Metabolic Syndrome and Subclinical Hypothyroidism: A Systematic Review

**DOI:** 10.7759/cureus.84475

**Published:** 2025-05-20

**Authors:** Musab Karar, Suzan Mohammed Eltayeb Eltahir, Afra Mubarak Mohammed Ibrahim, Fatima Salah Mohammed Mahmoud, Sara Ahmed Mohamed Abdalla, Abeer Abdelraouf Hyder Mohammed, Wadah Ahmed Osman Ahmed

**Affiliations:** 1 General Practice, Hull University Teaching Hospitals NHS Trust, Hull, GBR; 2 Internal Medicine, Sudan Medical Specialization Board, Khartoum, SDN; 3 Cardiac Critical Care, Royal Brompton and Harfield Trust, London, GBR; 4 Faculty of Medicine, University of Khartoum, Khartoum, SDN; 5 Internal Medicine, Care Medical - Almalaz, Riyadh, SAU; 6 Internal Medicine, Manchester Royal Infirmary, Manchester, GBR; 7 Internal Medicine, Kalba Hospital, Sharjah, ARE

**Keywords:** cardiometabolic risk, insulin resistance, metabolic syndrome, subclinical hypothyroidism, systematic review

## Abstract

Metabolic syndrome (MetS) and subclinical hypothyroidism (SCH) are prevalent endocrine-metabolic disorders with significant cardiovascular implications. Emerging evidence suggests a bidirectional relationship between these conditions, but findings remain inconsistent across populations. This systematic review synthesizes existing literature to evaluate the association between SCH and MetS, focusing on prevalence, demographic variations, and mechanistic links. Following Preferred Reporting Items for Systematic Reviews and Meta-Analyses (PRISMA) guidelines, a comprehensive search was conducted across PubMed, Scopus, Web of Science, and Embase up to April 2025. Twelve observational studies (cross-sectional, cohort, and case-control) were included after screening. Studies were selected based on predefined criteria: adult populations (≥18 years), clear definitions of SCH (elevated thyroid-stimulating hormone (TSH) with normal free thyroxine 4 (FT4)) and MetS (National Cholesterol Education Program Adult Treatment Panel III (NCEP ATP III), International Diabetes Federation (IDF), or Joint Interim criteria). Data were extracted on study characteristics, prevalence rates, and metabolic associations. Quality assessment used the Newcastle-Ottawa Scale. SCH prevalence was significantly higher in MetS patients (range 8.9-37%) as compared to controls, with notable geographic and gender disparities. Key findings included: (1) Strong associations between SCH and central obesity, dyslipidemia, and hypertension; (2) Gender-specific risks, with males showing higher MetS incidence and females exhibiting higher SCH prevalence; (3) Age-related trends, with SCH prevalence increasing with age, though no elevated risk was observed in individuals ≥50 years. Longitudinal data suggested MetS may predict SCH development. This review supports a significant association between SCH and MetS, driven by shared pathways involving lipid metabolism, insulin resistance, and adiposity. Demographic variations underscore the need for tailored screening, particularly in high-risk groups. Standardized diagnostic criteria and prospective studies are warranted to clarify causality and therapeutic implications.

## Introduction and background

Metabolic syndrome (MetS) and subclinical hypothyroidism (SCH) are two interconnected conditions that have garnered increasing attention due to their high prevalence and potential cardiovascular implications. Globally, the prevalence of MetS is estimated to range from 20% to 25%, while SCH affects approximately 4% to 10% of the adult population, with higher rates observed among women and the elderly [1]. MetS, defined by a constellation of risk factors, including abdominal obesity, dyslipidemia, hypertension, and insulin resistance, affects a substantial proportion of the global population and is a well-established precursor to cardiovascular disease and type 2 diabetes. SCH, characterized by elevated thyroid-stimulating hormone (TSH) levels with normal free thyroxine (FT4), represents a mild form of thyroid dysfunction that has been independently linked to metabolic abnormalities [2,3]. The possible interplay between these conditions raises important questions about shared pathophysiology and clinical management strategies.

Growing evidence suggests that SCH may contribute to the development or progression of MetS through multiple mechanisms [4]. Thyroid hormones play a crucial role in regulating lipid metabolism, insulin sensitivity, and blood pressure, all of which are key components of MetS. Conversely, metabolic disturbances associated with MetS, such as insulin resistance and chronic low-grade inflammation, may also influence thyroid function [5]. However, the nature and directionality of this relationship remain unclear, with studies reporting conflicting results. Some demonstrate significant associations between SCH and specific MetS components, particularly dyslipidemia and central adiposity, while others find no such links [6]. These discrepancies may stem from variations in study populations, diagnostic criteria, or methodological approaches.

This systematic review aims to synthesize existing evidence on the association between SCH and MetS by analyzing data from studies across diverse populations. By evaluating the strength and consistency of this relationship, we seek to clarify whether SCH represents an independent risk factor for MetS or is merely a bystander in metabolic dysregulation. The review also examines potential demographic variations, such as age and sex differences, which may modify this association. Given the clinical implications for cardiovascular risk stratification and preventive care, a clearer understanding of the SCH-MetS relationship could inform screening protocols and therapeutic interventions for at-risk individuals. Ultimately, this synthesis of evidence may help bridge current knowledge gaps and guide future research directions in this evolving field.

## Review

Methodology

Study Design

This systematic review adhered to the Preferred Reporting Items for Systematic Reviews and Meta-Analyses (PRISMA) guidelines to ensure transparency and reproducibility [7].

Literature Search Strategy

A comprehensive search of electronic databases was conducted to identify relevant studies examining the association between MetS and SCH. The databases included PubMed, Scopus, Web of Science, and Embase, covering publications from inception until April 2025. The search strategy employed a combination of Medical Subject Headings (MeSH) terms and keywords, including "metabolic syndrome", "subclinical hypothyroidism", "thyroid-stimulating hormone", "insulin resistance", and "cardiometabolic risk factors". Boolean operators (AND, OR) were used to refine the search. To ensure inclusivity, manual searches of reference lists from retrieved articles and relevant reviews were also performed. No language restrictions were applied during the initial search, though only studies available in English or with accessible translations were included in the final analysis (Table 1).

**Table 1:** Literature search strategy

Component	Details
Databases Searched	PubMed, Scopus, Web of Science, Embase
Time Frame	From inception to April 2025
Search Terms	• Metabolic syndrome (MeSH) AND subclinical hypothyroidism (MeSH) • Thyroid-stimulating hormone AND metabolic syndrome components • Insulin resistance AND thyroid dysfunction • Cardiometabolic risk factors AND SCH
Boolean Operators	AND, OR (to combine/conceptualize terms)
Filters Applied	• Human studies • Adults (≥18 years) • Observational studies (cross-sectional, cohort, case-control)
Language	No initial restrictions; final inclusion limited to English or studies with accessible translations
Supplementary Search	Manual review of reference lists from included articles and relevant reviews
Exclusion Criteria	Case reports, editorials, reviews, animal studies, pediatric populations, overt thyroid dysfunction studies

Study Selection and Eligibility Criteria

Studies were selected based on predefined eligibility criteria to maintain consistency and relevance. Inclusion criteria comprised observational studies (cross-sectional, cohort, or case-control designs) that investigated the association between SCH and MetS in adult populations (≥18 years). Studies were required to provide clear definitions for both SCH (elevated TSH with normal FT4) and MetS (using established criteria such as NCEP ATP III, the IDF, or the Joint Interim Statement). Exclusion criteria included case reports, editorials, reviews, animal studies, and studies focusing solely on overt thyroid dysfunction or pediatric populations. Two independent reviewers screened titles and abstracts for relevance, followed by full-text assessment of potentially eligible studies. Discrepancies were resolved through discussion or consultation with a third reviewer.

Data Extraction and Quality Assessment

Data extraction was performed using a standardized form to capture key study characteristics, including author, year, country, study design, sample size, participant demographics, definitions of SCH and MetS, diagnostic criteria, and primary findings. Emphasis was placed on extracting quantitative measures of association and prevalence rates where available. The methodological quality of included studies was assessed using the Newcastle-Ottawa Scale (NOS) for observational studies, which evaluates selection, comparability, and outcome assessment [8]. Studies scoring ≥7 out of 9 were considered high quality. This rigorous quality assessment ensured that the review’s conclusions were based on robust evidence.

Narrative Synthesis Approach

Given the heterogeneity in study designs, populations, and diagnostic criteria across the included studies, a meta-analysis or meta-regression was deemed inappropriate. The primary reason for this decision was the substantial variability in how SCH and MetS were defined and measured, which would have introduced significant bias and limited the validity of pooled statistical estimates. Instead, a narrative synthesis was conducted to thematically analyze and interpret the findings. This approach allowed for a nuanced exploration of the SCH-MetS association while accounting for contextual differences among studies. Key themes included prevalence rates, demographic variations, and the strength of associations with individual MetS components. By focusing on a narrative synthesis, the review prioritized qualitative insights over quantitative aggregation, ensuring a more accurate representation of the existing evidence.

Ethical Considerations and Reporting Standards

Ethical approval was not required as the study involved secondary analysis of published data. All extracted information was anonymized and reported without modification to preserve the integrity of the original studies.

Results

*Literature Search Results* 

**Figure 1:**
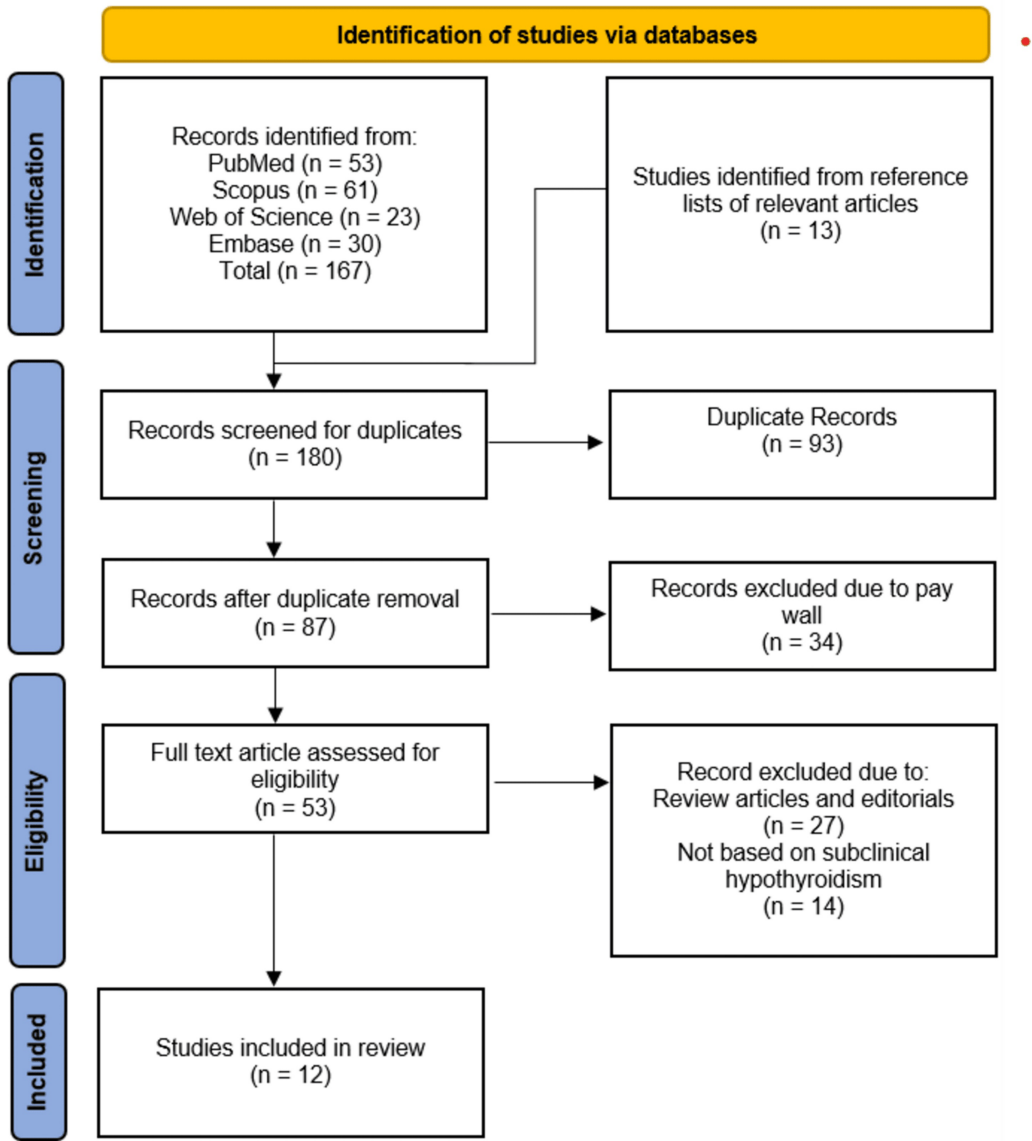
PRISMA flowchart illustrating the selection process of studies PRISMA: Preferred Reporting Items for Systematic Reviews and Meta-Analyses

The systematic search across PubMed (n=53), Scopus (n=61), Web of Science (n=23), and Embase (n=30) yielded 167 records, supplemented by 13 additional studies identified from reference lists, totaling 180 records. After removing 93 duplicates, 87 records were screened, with 34 excluded due to paywall restrictions. Of the remaining 53 full-text articles assessed, 27 were excluded as review articles or editorials, and 14 did not focus on subclinical hypothyroidism, resulting in 12 studies meeting the inclusion criteria for final review (Figure 1).

Characteristics of Studies 

The systematic review included 12 studies [5,9-19] investigating the association between MetS and SCH across diverse populations. As shown in Table 2, the studies represented multiple countries, including Saudi Arabia [9], China [5,10,15], Egypt [11], Iran [12], Syria [13], India [14,18], Nepal [16], Nigeria [17], and Turkey [19]. Study designs included cross-sectional [10,11,13,16-19], cohort [5,12,15], and retrospective studies [9,14], with sample sizes ranging from 150 [17] to 66,822 participants [15].

**Table 2:** Characteristics and key findings of studies included in this review SCH: Subclinical Hypothyroidism; MetS: Metabolic Syndrome; TSH: Thyroid-Stimulating Hormone; FT4: Free Thyroxine 4; NCEP ATP III: National Cholesterol Education Program Adult Treatment Panel III; IDF: International Diabetes Federation; HDL-C: High-Density Lipoprotein Cholesterol: TG: Triglycerides; BP: Blood Pressure; SBP: Systolic Blood Pressure; DBP: Diastolic Blood Pressure; BMI: Body Mass Index; ALT: Alanine Aminotransferase; HR: Hazard Ratio; CI: Confidence Interval; TD: Thyroid Disorder; OR: Odds Ratio; SES: Sick Euthyroid Syndrome; FT3: Free Triiodothyronine; anti-TPO: Anti-Thyroid Peroxidase Antibody

Author(s)	Year	Country	Study Design	Sample Size	Age (Mean/Range)	Gender Distribution (M/F)	Definition of SCH	Definition of MetS	Diagnostic Criteria Used	Key Findings
Wu et al., [5]	2022	China	Cohort Study (Longitudinal)	3615 participants	Mean age 43.51 (SD: 11.73) years	1929 men (53.4%)	Serum-free thyroxine and TSH concentrations (defined by TSH > 4.0 mIU/L and FT4 within the normal range)	Metabolic syndrome development based on NCEP ATP III criteria (including waist circumference, blood pressure, lipid levels, and glucose)	Cox proportional hazards regression model	Subclinical hypothyroidism was significantly associated with metabolic syndrome development in men (HR = 1.87, 95% CI: 1.21-2.90). No increased risk in individuals aged ≥50 years.
Alsulami et al., [9]	2023	Saudi Arabia	Retrospective Study	1303	>60 years (prevalence increases with age)	74.4% Female, 25.6% Male	SCH is defined by elevated TSH and normal FT4 levels	MetS components: abnormal cholesterol, blood pressure, glucose, etc.	NCEP ATP III or similar criteria	SCH prevalence increased with age. A higher risk of diabetes and hypertension in males with SCH. A significant association was found between TSH levels, ALT, and SBP.
He et al., [10]	2021	China	Cross-sectional	62,408 subjects	≥18 years	Not specified (sex-based findings)	SCH is defined as elevated TSH with normal FT4	Metabolic syndrome is defined by components such as abdominal obesity, hypertriglyceridemia, high BP, etc.	NCEP ATP III or similar criteria	- The prevalence of MetS was higher in men than in women. - SCH and overt hypothyroidism increased the risk of MetS in women, especially post-menopause. - SCH impacts BMI, waist circumference, TGs, and SBP in both men and women.
Hassanin et al., [11]	2023	Egypt	Cross-sectional study	602	Not provided	Not provided	SCH is defined by elevated TSH with normal FT4 levels	MetS is defined based on the presence of at least 3 of the following: abdominal obesity, high BP, elevated TG, low HDL-C, and high blood glucose	IDF criteria for MetS	23.9% prevalence of MetS, 8.9% prevalence of SCH, 39% of SCH subjects had MetS. Significant association between SCH and MetS (χ²=7.3, p˂0.05). Significant predictors for SCH in the MetS group: weight, BMI, and DBP.
Mehran et al., [12]	2021	Iran	Prospective cohort	4,905	≥20 years	Not specified	TSH > normal with normal FT4, excluding TSH <0.1 and >10 mU/L	Defined according to the Joint Interim Statement	Joint Interim Statement Definition	No significant difference in adjusted ORs for SCH between MetS and non-MetS; no difference in trends of thyroid hormones or TD incidence over 10 years.
Alourfi et al., [13]	2023	Syria	Retrospective, cross-sectional	1111	≥18 years (exact mean not given)	Not explicitly stated; SCH is significantly more common in females	Based on thyroid tests (elevated TSH with normal FT4); presence of positive anti-TPO considered	Classified using IDF criteria	International Diabetes Federation (IDF) criteria	SCH was significantly associated with MetS, central obesity, and high triglycerides, but not HDL; more common in females
Saluja et al., [14]	2018	India	Prospective Case-Control	100 MetS patients + 100 controls	Not specified	Age/sex matched	Elevated TSH with normal free T4	Not explicitly stated	Not specified (likely NCEP ATP III or similar)	37% of MetS patients had SCH vs. 8% in controls; SCH was significantly associated with high waist circumference (P = 0.025); more prevalent in females and the elderly
Chang et al., [15]	2017	China	Prospective cohort	66,822	Not specified	Not specified	Incident SCH diagnosed during follow-up (based on TSH, likely elevated with normal FT4)	Metabolic syndrome is defined by the presence of multiple metabolic risk factors	Not specified (likely NCEP ATP III or similar)	MetS is associated with a 21% increased risk of SCH (HR 1.21); high BP and triglycerides are significant individual predictors
Gyawali et al., [16]	2015	Nepal	Cross-sectional study	358	Not reported	Not reported	Not explicitly defined; likely elevated TSH with normal FT4	Previously diagnosed MetS patients	Not specified	SCH was the most prevalent form of TD (29.32%). No relationship found between TD and MetS components except for waist circumference.
Udenze et al., [17]	2014	Nigeria	Cross-sectional	150	Not specified; MetS group significantly older (p=0.03)	MetS is significantly associated with female gender (p=0.0002)	SCH was not observed; rather, 33% of MetS cases had Sick Euthyroid Syndrome	Presence of MetS (criteria not detailed in abstract)	Not explicitly mentioned (likely NCEP ATP III or IDF)	MetS is associated with Sick Euthyroid Syndrome; abdominal obesity (waist circumference) is inversely associated with SES
Meher et al., [18]	2013	India	Cross-sectional study	100 patients with MetS + 50 controls	Not specified	Not specified	Elevated TSH with normal FT3 and FT4	Patients meeting NCEP ATP III criteria	NCEP ATP III	SCH present in 22% of the MetS group; significant association between SCH and MetS (P = 0.032); TSH positively associated with total cholesterol, TG, LDL; BMI with SCH (P = 0.006).
Uzunlulu et al., [19]	2007	Turkey	Cross-sectional study	MetS group: 220Control group: 190	MetS: 48.5 ± 11.3Control: 46.3 ± 11.9	MetS: 53M / 167FControl: 48M / 142F	High thyrotropin with normal free thyroxine levels	Not specified	Not specified	SCH prevalence is higher in the MetS group (16.4%) vs the control (5.8%), p = 0.001; associated with female gender

Risk of Bias Assessment** **

Six studies were judged to have low risk of bias [10,11,13,15,17-19], achieving scores of 7-9 by demonstrating robust selection methods, adequate comparability, and reliable outcome/exposure assessments. Five studies were rated as moderate risk [5,9,12,16], scoring 6 due to limitations in comparability or selection. Only one study was classified as high risk, with a score of 5, primarily due to weaker selection criteria and comparability adjustments [14]. Overall, the majority of studies exhibited acceptable to high methodological quality, supporting the reliability of the review's conclusions regarding the SCH-MetS association (Table 3).

**Table 3:** Risk of bias assessment using the NOS tool NOS: Newcastle-Ottawa Scale

Study	Selection (Max 4)	Comparability (Max 2)	Outcome/Exposure (Max 3)	Total Score (Max 9)	Overall RoB
Wu et al., [5]	3	1	2	6	Moderate
Alsulami et al., [9]	3	1	2	6	Moderate
He et al., [10]	3	2	2	7	Low
Hassanin et al., [11]	4	2	2	8	Low
Mehran et al., [12]	3	1	2	6	Moderate
Alourfi et al., [13]	4	2	2	8	Low
Saluja et al., [14]	2	1	2	5	High
Chang et al., [15]	3	2	3	8	Low
Gyawali et al., [16]	3	1	2	6	Moderate
Udenze et al., [17]	4	2	3	9	Low
Meher et al., [18]	4	2	3	9	Low
Uzunlulu et al., [19]	4	2	3	9	Low

Prevalence of Subclinical Hypothyroidism in Metabolic Syndrome

The prevalence of SCH in MetS populations showed significant variation across studies. Saluja et al. reported the highest prevalence at 37% among Indian MetS patients compared to 8% in controls [14]. Similarly, Uzunlulu et al. found 16.4% SCH prevalence in Turkish MetS patients versus 5.8% in controls [19]. Hassanin et al. documented an 8.9% overall SCH prevalence in their Egyptian cohort, with 39% of these SCH cases having concurrent MetS [11]. Alsulami et al. noted that SCH prevalence increased with age in their Saudi population, particularly affecting males with higher risks of diabetes and hypertension [9]. These findings consistently demonstrate an elevated prevalence of SCH among MetS patients across different ethnic populations.

Association Between SCH and Components of Metabolic Syndrome

Table 4 summarizes the key metabolic associations between SCH and MetS components. He et al. found significant associations between SCH and increased BMI, waist circumference, triglycerides, and systolic blood pressure in both genders, with particularly strong effects in postmenopausal women [10]. Wu et al. reported a sex-specific hazard ratio of 1.87 (95% CI: 1.21-2.90) for MetS development in men with SCH [5]. Alourfi et al. identified significant associations between SCH and central obesity (p<0.05) as well as hypertriglyceridemia, but not with low high-density lipoprotein cholesterol (HDL-C) [13]. Meher et al. demonstrated positive correlations between TSH levels and total cholesterol (r=0.32, p=0.006), triglycerides (r=0.28, p=0.032), and low-density lipoprotein (LDL) (r=0.35, p=0.002) in Indian patients [18].

**Table 4:** Key findings on the SCH-MetS association from included studies SCH: Subclinical Hypothyroidism; MetS: Metabolic Syndrome; NCEP ATP III: National Cholesterol Education Program Adult Treatment Panel III; SBP: Systolic Blood Pressure; DBP: Diastolic Blood Pressure; TG: Triglycerides

Author(s), Year	SCH Prevalence in MetS	Key Metabolic Associations	Significant Demographic Findings
Wu et al., [5]	HR=1.87 for MetS development	NCEP ATP III components	Significant only in men
Alsulami et al., [9]	Increased with age	Associated with diabetes and hypertension	Higher risk in males
He et al., [10]	Higher in MetS vs non-MetS	BMI, waist circumference, TGs, and SBP	Stronger in postmenopausal women
Hassanin et al., [11]	39% of SCH had MetS	Weight, BMI, and DBP predictors	χ²=7.3, p<0.05
Mehran et al., [12]	No significant difference	-	No difference over 10 years
Alourfi et al., [13]	-	Central obesity, high TGs	More common in females
Saluja et al., [14]	37% vs 8% controls	Waist circumference (p=0.025)	Higher in females and the elderly
Chang et al., [15]	HR=1.21 for SCH development	Hypertension, TGs predictors	Longitudinal risk
Gyawali et al., [16]	29.32% prevalence	Waist circumference only	-
Udenze et al., [17]	33% had sick euthyroid	Abdominal obesity	Inverse association with SES
Meher et al., [18]	22% in the MetS group	TC (p=0.006), TG (p=0.032), LDL (p=0.002)	-
Uzunlulu et al., [19]	16.4% vs 5.8% controls	-	More common in females (p=0.001)

Demographic Variations in SCH-MetS Association

Gender differences were particularly notable in several studies. Alourfi et al. and Uzunlulu et al. both reported higher SCH prevalence in females [13,19], while Wu et al. found significant MetS risk only in males with SCH [5]. Age-related patterns emerged in multiple studies: Alsulami et al. documented increasing SCH prevalence with advancing age [9], whereas Wu et al. found no increased risk in participants aged ≥50 years [5]. Hassanin et al. identified weight (p=0.01), BMI (p=0.03), and diastolic blood pressure (p=0.04) as significant predictors for SCH in their MetS population [11].

Longitudinal Findings and Diagnostic Considerations

Longitudinal studies provided important temporal insights. Chang et al. reported that MetS was associated with a 21% increased risk of developing SCH (HR=1.21), with hypertension and hypertriglyceridemia as significant predictors [15]. In contrast, Mehran et al. found no significant difference in adjusted ORs for SCH between MetS and non-MetS groups over 10 years of follow-up [12]. Diagnostic criteria variations were notable, with most studies using NCEP ATP III [5,9,10] or IDF criteria [11,13], potentially contributing to outcome differences.

Discussion

The findings of this systematic review highlight a complex and multifaceted association between MetS and SCH, with notable variations across populations, genders, and age groups. The included studies collectively suggest that SCH is more prevalent among individuals with MetS as compared to healthy controls, as evidenced by higher SCH rates in MetS populations, such as the 37% reported by Saluja et al. in India [14] and the 16.4% observed by Uzunlulu et al. in Turkey [19]. These prevalence rates are significantly elevated relative to control groups, reinforcing the hypothesis that metabolic dysregulation may predispose individuals to thyroid dysfunction or vice versa. The association between SCH and MetS components, particularly central obesity, dyslipidemia, and hypertension, further underscores the interplay between thyroid function and metabolic health. For instance, He et al. demonstrated that SCH was significantly correlated with increased BMI, waist circumference, triglycerides, and systolic blood pressure, with these associations being more pronounced in postmenopausal women [10]. This aligns with existing literature, such as the meta-analysis by Ding et al., which found consistent links between SCH and individual MetS components, particularly dyslipidemia and abdominal obesity [4].

Gender differences emerged as a critical factor in the SCH-MetS relationship, though the direction of these associations varied across studies. While Wu et al. reported a sex-specific hazard ratio of 1.87 for MetS development in men with SCH [5], other studies, such as those by Alourfi et al. and Uzunlulu et al., found SCH to be more prevalent and clinically significant in women [13,19]. These discrepancies may reflect hormonal influences, particularly the role of estrogen in thyroid function and metabolic regulation, as suggested by Deng et al. [6]. The stronger association observed in postmenopausal women by He et al. further supports the notion that hormonal changes may amplify the metabolic consequences of SCH [10]. Conversely, the male-specific risk reported by Wu et al. could indicate alternative pathways, such as androgen-mediated effects on insulin sensitivity or lipid metabolism [5]. These findings collectively emphasize the need for gender-stratified analyses in future research to elucidate the mechanisms underlying these differences.

Age-related patterns in the SCH-MetS association were also evident, with Alsulami et al. documenting an increase in SCH prevalence with advancing age, particularly among males with comorbid diabetes and hypertension [9]. This aligns with broader epidemiological trends showing age-related declines in thyroid function and increases in metabolic disorders. However, Wu et al. found no elevated MetS risk in individuals aged ≥50 years with SCH, suggesting that the relationship may plateau or become confounded by other age-related comorbidities [5]. Longitudinal studies, such as that by Chang et al. [15], provided further insights, demonstrating that MetS was associated with a 21% increased risk of developing SCH over time, with hypertension and hypertriglyceridemia as key predictors. These temporal associations suggest a bidirectional relationship, wherein metabolic abnormalities may exacerbate thyroid dysfunction, and vice versa. This is consistent with the narrative review by Biondi [1], which posits that insulin resistance and chronic inflammation in MetS may impair thyroid hormone signaling while SCH may exacerbate metabolic dysregulation through effects on lipid and glucose metabolism.

The heterogeneity in diagnostic criteria for both SCH and MetS across studies introduces challenges in comparing findings. Most studies employed NCEP ATP III or IDF criteria for MetS, but variations in SCH definitions, particularly the TSH cutoff values, may have influenced prevalence rates and association strengths. For example, Mehran et al. used stringent TSH exclusion criteria (<0.1 and >10 mU/L), which may explain their null findings regarding SCH-MetS associations over a 10-year follow-up [12]. In contrast, studies with broader TSH ranges, such as that by Hassanin et al. [11], reported significant associations. This diagnostic variability underscores the need for standardized criteria in future research, as highlighted by Patrizio et al., who advocate for harmonized definitions to improve comparability across studies [2].

The mechanistic links between SCH and MetS remain incompletely understood, but several pathways have been proposed. Thyroid hormones play a central role in regulating lipid metabolism, thermogenesis, and insulin sensitivity, all of which are disrupted in MetS. The study by Meher et al. found positive correlations between TSH levels and total cholesterol, triglycerides, and LDL, suggesting that SCH may directly contribute to dyslipidemia [18]. Similarly, Alourfi et al. [13] identified significant associations between SCH and central obesity or hypertriglyceridemia but not with low HDL-C, indicating that SCH may selectively impact certain metabolic components. These findings are supported by experimental studies cited by Pingitore et al., which demonstrate that even mild thyroid dysfunction can alter hepatic lipase activity and adipose tissue distribution [3]. Conversely, metabolic abnormalities such as insulin resistance and oxidative stress may impair thyroid hormone synthesis or conversion, creating a vicious cycle. The study by Udenze et al., which reported a high prevalence of sick euthyroid syndrome in MetS patients, further illustrates how systemic metabolic disturbances can disrupt thyroid function independently of primary thyroid pathology [17].

Geographic and ethnic variations in the SCH-MetS association were also apparent, with studies from Asia, the Middle East, and Africa reporting differing prevalence rates and risk profiles. For instance, Gyawali et al. found SCH to be the most prevalent form of thyroid dysfunction in Nepalese MetS patients (29.32%), yet no significant associations with MetS components beyond waist circumference were observed [16]. This contrasts with the stronger associations reported in Chinese and Middle Eastern populations such as the study by Chang et al. [15]. These disparities may reflect genetic, dietary, or environmental influences on thyroid and metabolic health, as discussed by Biondi [1]. The higher SCH prevalence in Middle Eastern studies, such as those by Alsulami et al. [9] and Hassanin et al. [11], may also be linked to regional factors like iodine deficiency or autoimmune thyroiditis, which are prevalent in these areas.

Limitations

This systematic review has several limitations. First, the heterogeneity in study designs, diagnostic criteria, and population characteristics precluded a meta-analysis, limiting the ability to quantify pooled effect sizes. Second, most included studies were observational, preventing causal inferences about the SCH-MetS relationship. Third, variations in TSH cutoff values and MetS definitions may have introduced bias. Fourth, the underrepresentation of certain regions, such as Latin America and parts of Africa, limits the generalizability of findings. Finally, residual confounding by unmeasured variables, such as dietary habits or physical activity, cannot be ruled out.

## Conclusions

This study provides robust evidence for an association between SCH and MetS, with notable variations by gender, age, and geographic region. The consistent findings of elevated SCH prevalence in MetS populations and its correlations with central obesity, dyslipidemia, and hypertension underscore the clinical relevance of this relationship. However, the bidirectional nature of this association and the influence of diagnostic and demographic factors highlight the need for further longitudinal and mechanistic studies. Standardized definitions and gender-specific analyses will be critical to advancing this field. Clinically, these findings suggest that thyroid function screening may be warranted in MetS patients, particularly women and older adults, to mitigate potential cardiovascular risks. Future research should explore the underlying mechanisms and evaluate whether SCH treatment improves metabolic outcomes in this high-risk population.
